# Clinical Evaluation and Cost-Effectiveness Analysis of Serum Tumor Markers in Lung Cancer

**DOI:** 10.1155/2013/195692

**Published:** 2013-09-19

**Authors:** Rong Wang, Guoqing Wang, Nan Zhang, Xue Li, Yunde Liu

**Affiliations:** ^1^School of Laboratory Medicine, Tianjin Medical University, No. 1 Guangdong Road, Hexi District, Tianjin 300203, China; ^2^Department of Clinical Laboratory, Tianjin Stomatological Hospital, Tianjin 300041, China

## Abstract

The detection of serum tumor markers is valuable for the early diagnosis of lung cancer. Tumor markers are frequently used for the management of cancer patients. However, single markers are less efficient but marker combinations increase the cost, which is troublesome for clinics. To find an optimal serum marker combination panel that benefits the patients and the medical management system as well, four routine lung cancer serum markers (SCCA, NSE, CEA, and CYFRA21-1) were evaluated individually and in combination. Meanwhile, the costs and effects of these markers in clinical practice in China were assessed by cost-effectiveness analysis. As expected, combinations of these tumor markers improved their sensitivity for lung cancer and different combination panels had their own usefulness. NSE + CEA + CYFRA21-1 was the optimal combination panel with highest Youden's index (0.64), higher sensitivity (75.76%), and specificity (88.57%), which can aid the clinical diagnosis of lung cancer. Nevertheless, the most cost-effective combination was SCCA + CEA, which can be used to screen the high-risk group.

## 1. Introduction

Lung cancer has the highest incidence and mortality of any cancer worldwide. In 2008, 1.61 million new cases were reported, and 1.38 million deaths were attributed to lung cancer. The highest rates are in Europe and North America. In contrast to the declining mortality rate in men, lung cancer mortality rates in women have been rising over the recent decades [1]. In China, lung cancer has the highest incidence, and it is the leading cause of mortality of all cancers. This cancer is increasing at a rapid rate, and both incidence and mortality are steadily growing. China will drive up global rates of lung cancer in the foreseeable future [2].

Lung cancer patients often do not exhibit specific symptoms, particularly in early stages. Therefore, the majority of lung cancer patients are diagnosed at an advanced stage, which undermines their effective treatment. Currently, conventional diagnostic tests such as chest radiographs, computed tomography (CT) scans, and fiber optic bronchoscopy (FB) are not sensitive enough for effective early detection. Meanwhile, the benign pulmonary nodules and malignant tumors cannot be distinguished by imaging methods currently [3, 4]. Whereas, the pathological and cytological detections needed to obtain biopsy samples are invasive and difficult to repeat. Serum tumor markers are proteins that can be found in the blood, and their higher-than-normal concentrations have resulted in their widespread use in oncology [5, 6].

Early studies have illustrated the significance of serum tumor markers in the detection, prognosis, and follow-up of lung cancer [7–9]. Serum tumor markers such as carcinoembryonic antigen (CEA), squamous carcinoma antigen (SCCA), neuron specific enolase (NSE), and cytokeratin fragment (CYFRA21-1) have been investigated in patients with lung cancer. SCCA is a tumor-associated antigen originally isolated from squamous cell carcinoma of the uterine cervix [10]. Serum antigen levels have been used to follow the tumor status of squamous cell carcinomas, including those of the head and neck, oral cavity, esophagus, and lung [6]. NSE is the *γγ* dimer (isoenzyme) of enolase, which was first found in brain tissue extract and has been shown to be present in neuroendocrine cells and tumors [11]. NSE is a selective marker for small-cell carcinoma [12]. CEA was first identified by GOLD and FREEDMAN in 1965 as an antigen specific for digestive tract adenocarcinomas [13]. In comparative studies [14–16], CYFRA 21-1 has proven to be the marker of choice in nonsmall cell lung cancer and also exhibits independent prognostic value [17]. Serum biomarkers are useful for physicians to screen, diagnose, and treat lung cancer.

 However, the diagnostic value of a single marker is limited by its sensitivity and specificity [12, 18]. Therefore, combination marker panel is frequently chosen in the clinic. However, combination panel has increased costs. China is a developing country with a large population where nearly 70% of the people live in poor rural areas. Hence, the cost-effectiveness of each assay is an important consideration.

Aims of this study were (1) to further evaluate the clinical value of four common serum markers (SCCA, NSE, CEA, and CYFRA21-1) in our cohort and (2) to find an optimal serum marker combination panel that benefits both patients and the medical insurance system. 

## 2. Materials and Methods

### 2.1. Patient Population

Three groups of people were selected between March 2008 and December 2008 from the Tianjin Medical University Cancer Institute and Hospital. The first group comprised lung cancer patients. The diagnosis of each patient was confirmed by clinical outcome, imaging diagnosis and histological examinations. Stage and histological classification were evaluated according to the World Health Organization (WHO) 1999 lung cancer classification. All samples were collected before treatment. The second group was composed of patients with benign pulmonary diseases. In-patients with pneumonia, pleural effusion, bronchiectasia, and pulmonary abscess diagnoses were randomly selected. Patients were confirmed by routine standard diagnostic methods or histological examination, those patients with a history of malignant disease, digestive or kidney disease, or two or more concomitant lung diseases were excluded. The third group served as the healthy control group. Healthy people who took a physical examination and all the examination in the normal range were included, except for those with a family history of lung cancer. Detailed patient characteristics are described in Table 1.

### 2.2. Sample Collection and Detection

A 3 mL fasting venous blood sample was collected from each patient in the morning into a sterile tube. The samples were then centrifuged at 2,500 rpm for 20 min. The serums were stored at +4°C and at −70°C on the longterm. The SCCA concentration was determined by a microparticle enzyme immunoassay using Abbott reagent sets (Abbott, USA) and measured by a chemical luminescence analyzer (ARCHITECT i2000SR, Abbott, USA). The NSE, CEA, and CYFRA21-1 concentrations were detected by electrochemical luminescence (Roche Diagnostics, Shanghai, China). According to each manufacturer's recommendations, the positive cut-off values for each marker were 1.5 ng/mL for SCCA, 5.0 ng/mL for CEA, 3.3 ng/mL for CYFRA21-1, and 15.2 ng/mL for NSE. A positive sample was considered to be a sample with at least one positive serum marker in the marker combination panel.

### 2.3. Cost-Effectiveness Analysis

The cost-effectiveness of the combination marker panel was evaluated in lung cancer group. The effectiveness is determined by the tumor marker sensitivity, and the cost depends on the expense that patients incur for the detection. According to the charge fee in the third class A level hospital in Tianjin, the cost for SCCA detection was ¥77, and detections for the other three markers (NSE, CEA, CYFRA21-1) were each ¥100.

### 2.4. Statistical Analysis

Statistical analysis was performed using SPSS Statistics 19.0 (SPSS, Inc., Chicago, IL). The association between serum markers and lung cancer characteristics including stage and histological classification were compared by Student's *t*-test. The data were described by means ± standard deviation. The sensitivity, specificity, and Youden's index of single markers and combination markers were calculated. Receiver operation characteristic (ROC) curves were used to estimate the diagnostic efficiency of each marker. Three levels are stratified into no diagnostic value (<0.5), lower accuracy (0.5–0.7), higher accuracy (0.7–0.9), and highest accuracy (>0.9). Based on the statistics, the accuracy of the diagnostic assay is enhanced as the area under the ROC curve increases [19, 20]. A similar trend is exhibited by Youden's index [21]; the accuracy of the diagnostic strength increases with the index. Cost-effectiveness was analyzed by a cost-benefit ratio. 

## 3. Results

### 3.1. Comparison of the SCCA, NSE, CEA, and CYFRA21-1 Concentrations in the Lung Cancer Group, Benign Lung Disease Group, and Healthy Control Group

Table 2 showed that the four markers were more abundant in the lung cancer group than in the healthy control group; NSE, CEA, and CYFRA21-1 were dramatically increased (*P* < 0.01). The concentrations of CEA and CYFRA21-1 were higher in the benign disease group than in the healthy group (*P* < 0.01). The SCCA concentration was not significantly different between the cancer and benign disease groups, but the remaining three markers were significantly different in the two groups. 

### 3.2. Comparison of the SCCA, NSE, CEA, and CYFRA21-1 Concentrations among Each Clinicopathological Factor in the Cancer Group

Table 3 showed a significant increase in the concentrations of SCCA and CYFRA21-1 in squamous cell carcinoma, an increased NSE concentration in small cell carcinoma, and an increased CEA concentration in adenocarcinoma (*P* < 0.05).

 With respect to TNM stage, only CEA was dramatically increased in stages III/IV when compared to stages I/II (*P* < 0.05), while no differences were observed in the concentrations of the SCCA, NSE, and CYFRA21-1 markers between the two stages. Similar to the trend in TNM stage, CEA demonstrated a higher concentration in the extensive disease group than in the limited disease. No significant difference was observed in the remaining three markers.

### 3.3. Comparison on ROC Curves of SCCA, NSE, CEA, and CYFRA21-1

**Figure 1:**
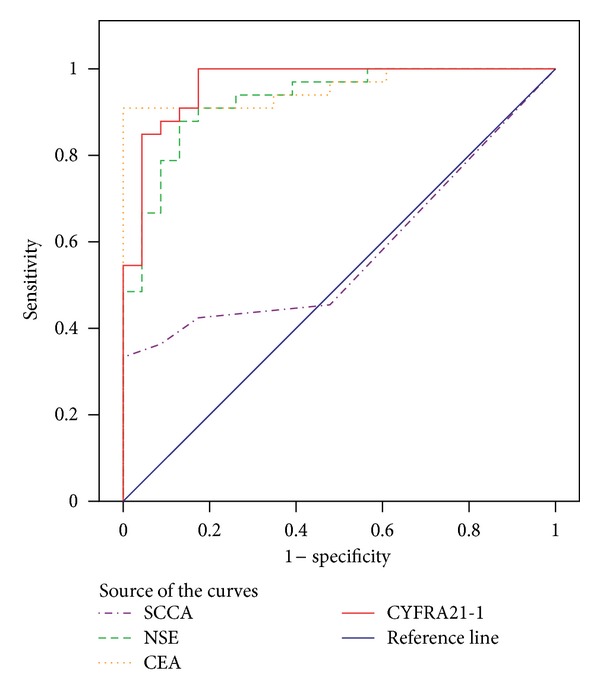
Receiver operation characteristic curves (ROCs) for the tumor markers in serum for the discrimination between lung cancer and healthy control. SCCA 0.578 ± 0.077 (95% CI 0.427~0.728), NSE 0.928 ± 0.034 (95% CI 0.861~0.994), CEA 0.957 ± 0.026 (95% CI 0.905~1.008), and CYFRA21-1 0.964 ± 0.023 (95% CI 0.919~1.01).

Figure 1 demonstrated that the sensitivities of NSE, CEA, and CYFRA21-1 (*P* < 0.05) were better than those of SCCA. The areas under the curves of NSE, CEA, and CYFRA21-1 were 0.928 ± 0.034, 0.957 ± 0.026, and 0.964 ± 0.023, respectively. 

### 3.4. Comparison of the Sensitivity, Specificity, and Youden's Index of the Four Markers Individually and in Combination

In descending order of individual sensitivity, the tumor markers were NSE (48.48%) > CYFRA21-1(46.21%) > CEA (45.45%) > SCCA (25.76%) (Table 4). The combination of tumor markers can improve the sensitivity, and the combination of NSE + CEA + CYFRA21-1 ranked the highest in Youden's index (0.64) with higher sensitivity (75.76%) and specificity (88.57%). 

### 3.5. Cost-Effectiveness Analysis

In the cost-effectiveness analysis, only the combination markers whose sensitivity exceeded 50% were included. The combination of SCCA and NSE was taken as the healthy control group because it exhibited the lowest sensitivity and the lowest cost.  Table 5 demonstrated that the most cost-effective combination was SCCA + CEA given that its cost was the lowest; furthermore, its cost was the lowest per 1% of sensitivity. When the cost of the assays was decreased by 10%, the cost of the SCCA + CEA combination was still the lowest as well as the ratio of cost to sensitivity. 

## 4. Discussion 

In this study, four common serum markers (SCCA, NSE, CEA, and CYFRA21-1) in lung cancer were evaluated individually and in combination. In addition to sensitivity and specificity, the ROC curve and Youden's index were applied, which are more accurate, effective, and comprehensive indexes for validation. Compared to SCCA, the markers NSE, CEA, and CYFRA21-1 were more accurate according to both higher Youden's indexes (0.40, 0.35, 0.43) and larger areas under ROC curves (0.928 ± 0.034, 0.957 ± 0.026, and 0.964 ± 0.023). However, the sensitivities of all four individual markers in our investigation were lower than 50%. Table 4 demonstrates that the combination of tumor markers is one way to improve their sensitivities. The combination of NSE + CEA + CYFRA21-1 might be an optimal choice due to the highest Youden's index (0.64) with higher sensitivity (75.76%) and specificity (88.57%). Furthermore, different combination panels can assist to differentiate histological subtype of lung cancer. The combination of SCCA, NSE, and CEA with the highest sensitivity (67.65%) and higher specificity (89.29%) can help in the diagnosis of AC; SCCA, CEA, and CYFRA21-1 (91.07% and 89.29%) can aid to the diagnosis of SCC; and SCCA, NSE, and CYFRA21-1 (75.00% and 88.57%) can assist in the diagnosis of SCLC. These results are associated to the unique function of each marker in previous reports.

 Enolase molecules in mammalian tissues are dimers composed of three immunologically distinct subunits (*α*, *β* and *γ*). The *γ* subunit, which has been designated as neuron-specific enolase (NSE), is highly concentrated in neurons, neuroendocrine cells, and neurogenic tumors [11]. NSE can be taken as a distinguishing marker between SCLC and NSCLC given that SCLC is a neurosecretion tumor that results in NSE expressing highly in SCLC patients [22, 23]. High serum levels of NSE in patients with suspicion of malignancy suggest the presence of SCLC with high probability. Previous studies have indicated that approximately 70% of 450 SCLC patients have elevated serum concentrations of NSE while only 14% of 190 NSCLC patients show this [6]. Consistent with this conclusion, the concentration of NSE in SCLC is higher than that in SCC and AC (*P* < 0.05) in our study. Contrary to our results, no difference was observed between LD and ED, high pretreatment values of NSE were noted in 38–71% of SCLC patients with LD and in 83–98% of those with ED and were summarized in Ferrigno's review [6].

Compared to NSE, the CEA, CYFRA21-1, and SCCA are more specific to NSCLC in our investigation. CEA is produced by the secretion cells of the normal adult gastrointestinal tract [24]. It is a marker for monitoring colon and rectal cancers. Recently, CEA has become the marker of choice for lung AC [25]. Meanwhile, several studies have reported increased CEA values in advanced bronchogenic cancers of various histological types [26, 27]. Generally, CEA levels vary in accordance with obvious changes in disease status, or they may precede their clinical recognition. Our data show that CEA has elevated levels (*P* < 0.05) and higher sensitivity (58.82%) in lung AC. Furthermore, it may have a role in monitoring therapy in advanced stages. In the present study, CEA is correlated with TNM stage and tumor invasion. In stages III/IV, CEA is observed at higher levels than in stages I/II. The same event occurs to the extensive lung cancer. However, the SCCA, NSE, and CYFRA21-1 do not show the correlation with TNM stage and extent of disease. In addition, the AUC for CEA in our study is higher than those in the literature where they range from 0.69 to 0.79 [28–31], which might be caused by samples in advanced stages or those including distant metastases.

CYFRA 21-1 is a sensitive tumor marker for NSCLC, particularly in squamous cell tumors. Because CYFRA 21-1 determines only fragments of cytokeratin 19, the test shows a higher specificity than tissue polypeptide antigen (TPA), which determines a mixture of cytokeratins 8, 18, and 19. CK-19 is a protein component of the intermediate filament protein in epithelial cells [32]. When epithelial cells transform into malignant cells, the keratin content is increased. Due to necrosis of tumor cells, the soluble fragment CYFRA21-1 of CK-19 is released into the blood. However, no organ tissue-specific and tumor-specific epithelial cytokeratins exist; therefore, it cannot be used as a tumor diagnosis indicator. However, CYFRA21-1 in serum will increase when epithelial cells transform into cancerous tumor cells, especially squamous epithelial cells of the lung and bladder transitional cells [32]. The results of this study indicate that the CYFRA21-1 concentration in the lung cancer group was significantly higher than that in the normal control and benign lung disease groups (*P* < 0.01), and the concentration in the benign lung disease group was significantly higher than that in the normal control group (*P* < 0.01). Therefore, its measurement may be helpful in the differential diagnosis of suspicious lung masses, but it can also be used as a good indicator for distinguishing between benign lung disease and normal group. Moreover, Table 3 shows that the expression of CYFRA21-1 in NSCLC was higher than that in SCLC. CYFRA21-1 and SCCA in SCC were more sensitive than those in AC and SCLC (*P* < 0.05) which is consistent with other reports [33, 34]. In descending order of CYFRA21-1 sensitivity, each pathological types are SCC (71.43%) > AC (29.41%) > SCLC (12.50%). This might be caused by the CK-19 expression in SCC and AC, while CK-18 is always expressed in SCLC. Taken together, CYFRA21-1 has a high diagnostic value in SCC.

SCCA is a purified subfraction of the tumor antigen. Elevated SCCA serum levels were found in many types of SCC, including the uterine cervix, bronchus, and nasopharynx [6, 10]. In 1988, Mino et al. [35] found higher SCCA serum levels in patients with lung squamous carcinoma compared to healthy subjects or those with benign pulmonary diseases. Our data are in partial agreement with this result; a higher level of SCCA is found in lung cancer, but it is only significantly different from the healthy control group and not different from the benign pulmonary group. Moreover, given that SCCA yielded the lowest sensitivity (25.76%) and the lowest Youden's index (0.20) in addition to exhibiting no significant difference from the control group by the ROC curve, SCCA may not be a good marker for lung cancer screening, but it might be useful as a marker for histological subtyping. However, because of its significantly reduced sensitivity, SCCA would preferably be used in combination with CYFRA 21-1, which is also specific for SCC. 

Although the combination of tumor markers can improve the sensitivity, the specificity will decrease with increasing sensitivity, meanwhile, the cost will increase as well. Presently, some hospitals and clinics prefer to take the four markers together (SCCA + NSE + CEA + CYFRA21-1). In reality, our results indicate that Youden's index and specificity of four marker panel are lower than three marker panel (NSE + CEA + CYFRA21-1) for the diagnosis of lung cancer. China is a developing country, and therefore, the optimal cost should be considered. The best marker combination panel is useful not only for promoting the efficiency of diagnosis, but also for reducing the economic load for the patient and health management department. Some reports [36–38] indicate that an analysis of cost-effectiveness is an appropriate evaluation of tumor marker combinations. In this study, we perform an analysis of cost-effectiveness for the tumor marker combinations. The results demonstrate that the cost of SCCA + CEA is the lowest per 1% sensitivity. However, many variables will affect the cost-effectiveness analysis. For instance, cost, discount rate, and depreciation of fixed assets may contribute to cost-effectiveness. If one of the variables varies, the result may change. Therefore, Δ*C*/Δ*E* (*C*: cost, *E*: effectiveness) is applied to evaluate the result due to the variables. With the advancement of laboratory medicine and instruments, the cost will decrease. We decreased the expense by 10%, and reassessed the tumor marker cost-effectiveness. The results remained unchanged. Hence, compared to other panels, the combination of SCCA and CEA is the best choice to implement screening program in high-risk group. 

## 5. Conclusions

Lung cancer is the most common and lethal malignant neoplasm in most of the western countries, and it is becoming one of the major health problems in undeveloped countries. The above data may explain several limitations in the clinical use of serum tumor markers. Although the four markers in our investigation are quick, objective, comparable, and reproducible, none of the single serum markers has sufficient sensitivity for the screening and diagnosis of lung cancer. Combination tumor markers could be a choice to improve the clinical effectiveness in the diagnosis of lung cancer. Furthermore, different combination panels have their own usefulness. In our investigation, the optimal marker panel with NSE, CEA, and CYFRA21-1 can assist to the diagnosis of lung cancer, and SCCA + NSE + CEA can help in the diagnosis of AC; SCCA + CEA + CYFRA21-1 can aid to the diagnosis of SCC; and SCCA + NSE + CYFRA21-1 can assist in the diagnosis of SCLC. In addition, from an economic viewpoint, SCCA + CEA might be a cost-effective combination for screening program. However, a large cohort validation and other tumor markers would be valuable research undertakings in the future.

## Figures and Tables

**Table 1:** Patient characteristics.

	Lung cancer group (132)	Pulmonary benign disease group (48)	Normal group (92)
Gender			
Male	88	28	48
Female	44	20	44
Age	28~81 (54.6)	22~82 (56.3)	18~82 (53.5)
Lung cancer			
Histology			
Adenocarcinoma	68		
Squamous cell lung cancer	56		
Small cell lung cancer	8		
NSCLC stages			
I/II	73		
III/IV	51		
SCLC stages			
Limited disease	3		
Extensive disease	5		
Pulmonary benign disease			
Pneumonia		14	
Pleural effusion		12	
Bronchiectasia		4	
Pulmonary abscess		2	
Phthisis		16	

**Table 2:** Concentrations of SCCA, NSE, CEA, and CYFRA21-1 in serum (ng/mL, $\overset{-}{x}\pm s$).

Group	Number	SCCA	NSE	CEA	CYFRA21-1
Lung cancer	132	1.73 ± 3.98*	19.45 ± 14.47^∗∗★^	17.81 ± 35.79^∗∗★★^	6.54 ± 7.36^∗∗★★^
Pulmonary benign disease	48	0.38 ± 0.55	10.88 ± 1.87	1.13 ± 0.24**	1.72 ± 0.83**
Healthy control	92	0.17 ± 0.10	9.09 ± 2.81	0.78 ± 0.30	1.03 ± 0.52

*P* value was calculated by Student's *t*-test *compared to healthy control, *P* < 0.05; **compared to healthy control, *P* < 0.01.

^★^compared to pulmonary benign disease, *P* < 0.05; ^★★^compared to pulmonary benign disease, *P* < 0.01.

**Table 3:** The relationship between SCCA, NSE, CEA, and CYFRA21-1 and the clinicopathological factors (ng/mL, $\overset{-}{x}\pm s$).

Clinicopathological characteristics	*N*	SCCA	NSE	CEA	CYFRA21-1
Histological classification					
Adenocarcinoma	68	0.22 ± 0.26	17.95 ± 8.30	30.76 ± 46.78*	4.00 ± 3.76
Squamous cell lung cancer	56	3.79 ± 5.56*	16.83 ± 5.66	4.49 ± 2.49	10.34 ± 9.38*
Small cell lung cancer	8	0.15 ± 0.07	50.80 ± 22.60*	1.81 ± 1.05	1.31 ± 0.30
NSCLC TNM stage					
I/II	73	2.59 ± 4.92	15.21 ± 4.53	7.44 ± 8.36	7.32 ± 9.01
III/IV	51	0.70 ± 1.03	21.94 ± 19.15	33.71 ± 50.04*	5.65 ± 3.99
SCLC stage					
Limited disease (LD)	3	2.06 ± 1.87	23.43 ± 16.74	7.27 ± 6.12	7.11 ± 5.23
Extensive disease (ED)	5	0.86 ± 0.79	25.02 ± 10.90	33.51 ± 29.63*	5.92 ± 3.80

*P* value was calculated by Student's *t*-test *P < 0.05.

**Table 4:** The sensitivity, specificity, and Youden's index of the four markers individually and in combination.

Tumor markers		Sensitivity (%)			Specificity (%)	Youden's index
	Adenocarcinoma	Squamous cell lung cancer	Small cell lung cancer	Lung cancer		
SCCA	7.35 (5/68)	50.00 (28/56)	12.50 (1/8)	25.76 (34/132)	94.29 (132/140)	0.20
NSE	47.06 (32/68)	50.00 (28/56)	50.00 (4/8)	48.48 (64/132)	91.43 (128/140)	0.40
CEA	58.82 (40/68)	35.71 (20/56)	0.00 (0/8)	45.45 (60/132)	89.29 (125/140)	0.35
CYFRA21-1	29.41 (20/68)	71.43 (40/56)	12.50 (1/8)	46.21 (61/132)	97.14 (136/140)	0.43
SCCA + NSE	47.06 (32/68)	67.86 (38/56)	62.50 (5/8)	56.82 (75/132)	91.43 (128/140)	0.48
SCCA + CEA	58.82 (40/68)	69.64 (39/56)	12.50 (1/8)	60.61 (80/132)	89.29 (125/140)	0.50
SCCA + CYFRA21-1	29.41 (20/68)	71.43 (40/56)	25.00 (2/8)	46.97 (62/132)	90.71 (127/140)	0.38
NSE + CEA	66.18 (45/68)	71.43 (40/56)	50.00 (4/8)	67.42 (89/132)	89.29 (125/140)	0.57
NSE + CYFRA21-1	52.94 (36/68)	78.57 (44/56)	62.50 (5/8)	64.39 (85/132)	88.57 (124/140)	0.53
CEA + CYFRA21-1	58.82 (40/68)	78.57 (44/56)	12.50 (1/8)	64.39 (85/132)	89.29 (125/140)	0.54
SCCA + NSE + CEA	67.65 (46/68)	75.00 (42/56)	62.50 (5/8)	70.45 (93/132)	89.29 (125/140)	0.60
SCCA + NSE + CYFRA21-1	52.94 (36/68)	78.57 (44/56)	75.00 (6/8)	65.15 (86/132)	88.57 (124/140)	0.54
SCCA + CEA + CYFRA21-1	58.82 (40/68)	91.07 (51/56)	25.00 (2/8)	70.45 (93/132)	89.29 (125/140)	0.60
NSE + CEA + CYFRA21-1	66.18 (45/68)	89.29 (50/56)	62.50 (5/8)	75.76 (100/132)	88.57 (124/140)	0.64
SCCA + NSE + CEA + CYFRA21-1	67.65 (46/68)	91.07 (51/56)	75.00 (6/8)	78.03 (103/132)	85.00 (119/140)	0.63

**Table 5:** Cost-effectiveness analysis of different combinations of tumor markers at the current cost and with a 10% decrease in cost.

Group	Cost (*C*)/¥	Effectiveness (*E*)/%	*C*/*E*	Δ*C*/Δ*E*
SCCA + NSE	177 (159.3)	56.82	3.12 (2.80)	0.00 (0.00)
SCCA + CEA	177 (159.3)	60.61	2.92 (2.63)	0.00 (0.00)
NSE + CEA	200 (180)	67.42	2.97 (2.67)	2.17 (1.95)
NSE + CYFRA21-1	200 (180)	64.39	3.11 (2.80)	3.04 (2.73)
CEA + CYFRA21-1	200 (180)	64.39	3.11 (2.80)	3.04 (2.73)
SCCA + NSE + CEA	277 (249.3)	70.45	3.93 (3.54)	7.34 (6.60)
SCCA + NSE + CYFRA21-1	277 (249.3)	65.15	4.25 (3.83)	12.00 (10.80)
SCCA + CEA + CYFRA21-1	277 (249.3)	70.45	3.93 (3.54)	7.34 (6.60)
NSE + CEA + CYFRA21-1	300 (270)	75.76	3.96 (3.56)	6.49 (5.84)
SCCA + NSE + CEA + CYFRA21-1	377 (339.3)	78.03	4.83 (4.35)	9.43 (8.49)

## List of Abbreviations

CEA: Carcinoembryonic antigen
NSE: Neuron-specific enolase
SCCA: Squamous cell carcinoma antigen
NSCLC: Non-small cell lung cancer
SCLC: Small cell lung cancer
SCC: Squamous cell carcinoma
AC: Adenocarcinoma
ED: Extensive disease
LD: Limited disease
ROC: Receiver operating characteristic
AUC: Area under ROC curve
TNM: TNM classification of malignant tumors.

## References

[1] Ferlay J, Shin H-R, Bray F, Forman D, Mathers C, Parkin DM (2010). Estimates of worldwide burden of cancer in 2008: GLOBOCAN 2008. *International Journal of Cancer*.

[2] Chang S, Dai M, Ren JS, Chen YH, Guo LW (2012). Estimates and prediction on incidence, mortality and prevalence of lung cancer in China in 2008. *Zhonghua Liu Xing Bing Xue Za Zhi*.

[3] Xie X, Zhao Y, Snijder RA (2013). Sensitivity and accuracy of volumetry of pulmonary nodules on low-dose 16- and 64-row multi-detector CT: an anthropomorphic phantom study. *European Radiology*.

[4] Henschke CI, Yankelevitz DF (2008). CT screening for lung cancer: update 2007. *Oncologist*.

[5] Pamies RJ, Crawford DR (1996). Tumor markers: an update. *Medical Clinics of North America*.

[6] Ferrigno D, Buccheri G, Biggi A (1994). Serum tumour markers in lung cancer: history, biology and clinical applications. *European Respiratory Journal*.

[7] de Cos Escuin JS, Hernandez JH (2004). Tumor markers and lung cancer. What’s new?. *Archivos de Bronconeumología*.

[8] Niewoehner DE, Rubins JB (2003). Clinical utility of tumor markers in the management of non-small cell lung cancer. *Methods in Molecular Medicine*.

[9] Viñolas N, Molina R, Galán MC (1998). Tumor markers in response monitoring and prognosis of non-small cell lung cancer: preliminary report. *Anticancer Research*.

[10] Kato H, Tamai K, Magaya T (1985). Clinical value of SCC-antigen, a subfraction of tumor antigen TA-4, in the management of cervical cancer. *Gan no Rinshos*.

[11] Cooper EH, Splinter TA, Brown DA, Muers MF, Peake MD, Pearson SL (1985). Evaluation of a radioimmunoassay for neuron specific enolase in small cell lung cancer. *British Journal of Cancer*.

[12] Jørgensen LGM, Osterlind K, Genollá J (1996). Serum neuron-specific enolase (S-NSE) and the prognosis in small-cell lung cancer (SCLC): a combined multivariable analysis on data from nine centres. *British Journal of Cancer*.

[13] Hammarström S (1999). The carcinoembryonic antigen (CEA) family: structures, suggested functions and expression in normal and malignant tissues. *Seminars in Cancer Biology*.

[14] Grenier J, Pujol JL, Guilleux F, Daures JP, Pujol H, Michel FB (1994). Cyfra 21-1, a new marker of lung cancer. *Nuclear Medicine and Biology*.

[15] Muraki M, Tohda Y, Iwanaga T, Uejima H, Nagasaka Y, Nakajima S (1996). Assessment of serum CYFRA 21-1 in lung cancer. *Cancer*.

[16] van der Gaast A, Schoenmakers CHH, Kok TC, Blijenberg BG, Cornillie F, Splinter TAW (1994). Evaluation of a new tumour marker in patients with non-small-cell lung cancer: Cyfra 21.1. *British Journal of Cancer*.

[17] Yan H-J, Wang R-B, Zhu K-L (2012). Cytokeratin 19 fragment antigen 21-1 as an independent predictor for definitive chemoradiotherapy sensitivity in esophageal squamous cell carcinoma. *Chinese Medical Journal*.

[18] Plebani M, Basso D, Navaglia F, de Paoli M, Tommasini A, Cipriani A (1995). Clinical evaluation of seven tumour markers in lung cancer diagnosis: can any combination improve the results?. *British Journal of Cancer*.

[19] McNeil BJ, Keeler E, Adelstein SJ (1975). Primer on certain elements of medical decision making. *The New England Journal of Medicine*.

[20] Hanley JA, McNeil BJ (1982). The meaning and use of the area under a receiver operating characteristic (ROC) curve. *Radiology*.

[21] Perkins NJ, Schisterman EF (2006). The inconsistency of “optimal” cutpoints obtained using two criteria based on the receiver operating characteristic curve. *American Journal of Epidemiology*.

[22] Satoh H, Ishikawa H, Kurishima K, Yamashita YT, Ohtsuka M, Sekizawa K (2002). Cut-off levels of NSE to differentiate SCLC from NSCLC. *Oncology Reports*.

[23] Ando S, Kimura H, Iwai N (2001). Optimal combination of seven tumour markers in prediction of advanced stage at first examination of patients with non-small cell lung cancer. *Anticancer Research*.

[24] Go VL, Ammon HV, Holtermuller KH, Krag E, Phillips SF (1975). Quantification of carcinoembryonic antigen like activities in normal human gastrointestinal secretions. *Cancer*.

[25] Foa P, Fornier M, Miceli R (1999). Tumour markers CEA, NSE, SCC, TPA and CYFRA 21.1 in resectable non-small cell lung cancer. *Anticancer Research*.

[26] Plavec G, Ninković M, Kozlovacki G, Lazarov A, Tatić V (2002). Tumor markers in pleural effusions in bronchogenic carcinoma and tuberculosis. *Vojnosanitetski Pregled*.

[27] Rasmuson T, Bjork GR, Damber L (1983). Tumor markers in bronchogenic carcinoma. An evaluation of carcinoembryonic antigen, tissue polypeptide antigen, placental alkaline phosphatase and pseudouridine. *Acta Radiologica*.

[28] Nisman B, Lafair J, Heching N (1998). Evaluation of tissue polypeptide specific antigen, CYFRA 21-1, and carcinoembryonic antigen in nonsmall cell lung carcinoma: does the combined use of cytokeratin markers give any additional information?. *Cancer*.

[29] Takada M, Masuda N, Matsuura E (1995). Measurement of cytokeratin 19 fragments as a marker of lung cancer by CYFRA 21-1 enzyme immunoassay. *British Journal of Cancer*.

[30] Bergman B, Brezicka F-T, Engstrom C-P, Larsson S (1993). Clinical usefulness of serum assays of neuron-specific enolase, carcinoembryonic antigen and CA-50 antigen in the diagnosis of lung cancer. *European Journal of Cancer A*.

[31] Moro D, Villemain D, Vuillez JP, Delord CA, Brambilla C (1995). CEA, CYFRA21-1 and SCC in non-small cell lung cancer. *Lung Cancer*.

[32] Stieber P, Dienemann H, Hasholzner U (1994). Comparison of CYFRA 21-1, TPA and TPS in lung cancer, urinary bladder cancer and benign diseases. *International Journal of Biological Markers*.

[33] Tomita M, Shimizu T, Ayabe T, Yonei A, Onitsuka T (2010). Prognostic significance of tumour marker index based on preoperative CEA and CYFRA 21-1 in non-small cell lung cancer. *Anticancer Research*.

[34] Lai R-S, Hsu H-K, Lu J-Y, Ger L-P, Lai N-S (1996). CYFRA 21-1 enzyme-linked immunosorbent assay: evaluation as a tumor marker in non-small cell lung cancer. *Chest*.

[35] Mino N, Iio A, Hamamoto K (1988). Availability of tumor-antigen 4 as a marker of squamous cell carcinoma of the lung and other organs. *Cancer*.

[36] Atherly AJ, Camidge DR (2012). The cost-effectiveness of screening lung cancer patients for targeted drug sensitivity markers. *British Journal of Cancer*.

[37] Hou J-X, Yang X-Q, Chen C, Jiang Q, Yang G-L, Li Y (2011). Screening the gastric cancer related tumor markers from multi-tumor markers protein chip with kappa coefficient and cost-effectiveness analysis. *Hepato-Gastroenterology*.

[38] McGhan WF, Rowland CR, Bootman JL (1978). Cost-benefit and cost-effectiveness: methodologies for evaluating innovative pharmaceutical services. *American Journal of Hospital Pharmacy*.

